# Supplementary material for: Cell-size dependent progression of the cell cycle creates homeostasis and flexibility of plant cell size
Source: Nat Commun. 2017 Apr 27;8:15060. doi: 10.1038/ncomms15060 (PMC5414177; doi:10.1038/ncomms15060)
Supplement: Supplementary Information — Supplementary Figures and Supplementary Tables [file ncomms15060-s1.pdf]

SUPPLEMENTAL INFORMATION

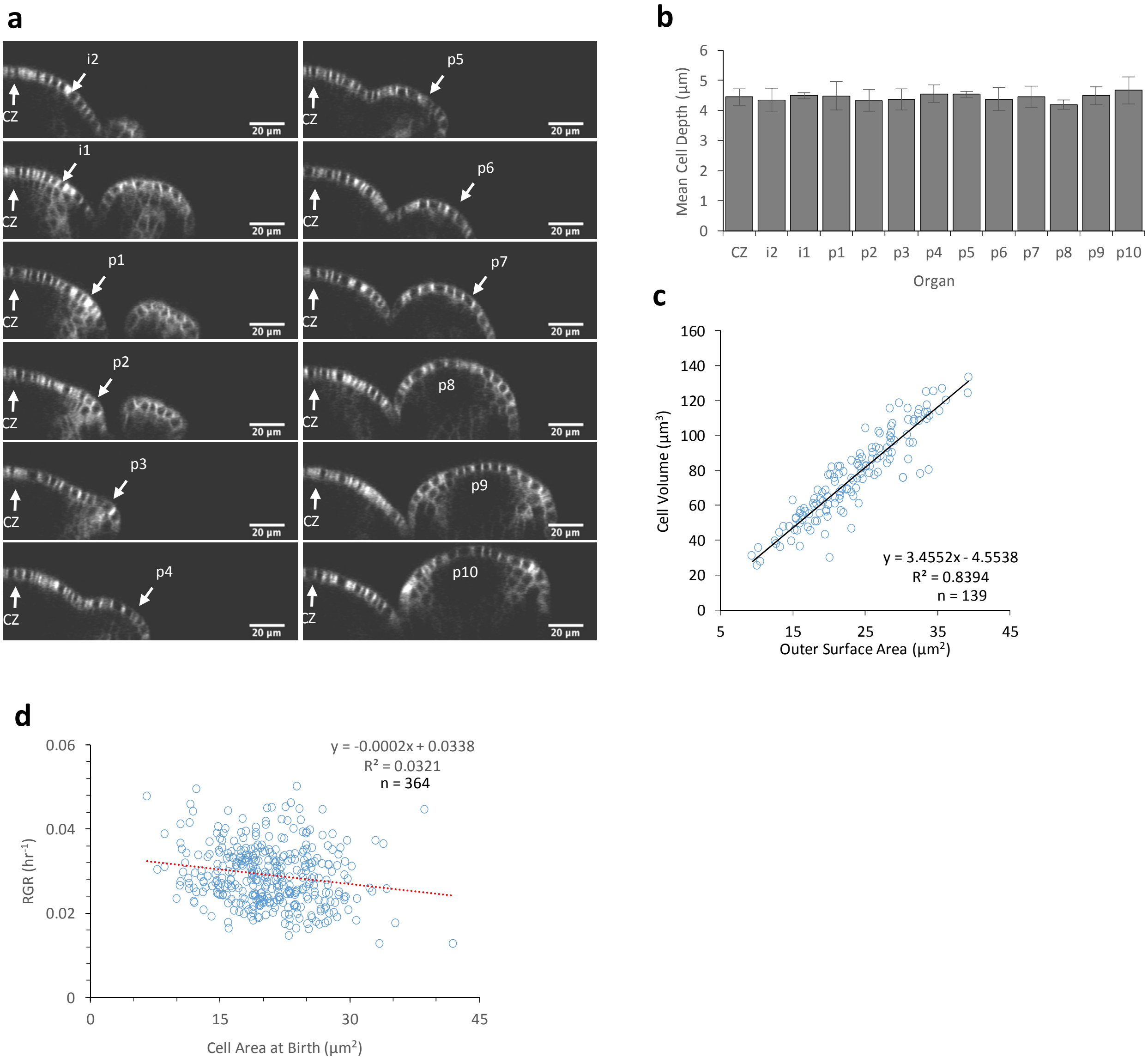

Supplementary Figure 1: Outer surface area of cells is a good proxy for cell volume.

a) Transverse sections of the SAM bisecting primordia of different developmental stages showing cell membranes of cells in the layer one of the meristem. Scale bars represent 20 µm.

b) Mean length of anticlinal cell walls during different developmental stages. Ten measurements were made per developmental stage. Error bars show standard deviation.

c) Relationship between outer cell surface area of cells measured in 2.5D using MorphoGraphX and cell volume measured in 3D using ImageJ Segmentation Editor, a manual segmentation tool.

d) Relationship between cell size and RGR over a 24 hour period of cells from the central region in between outgrowing primordia.

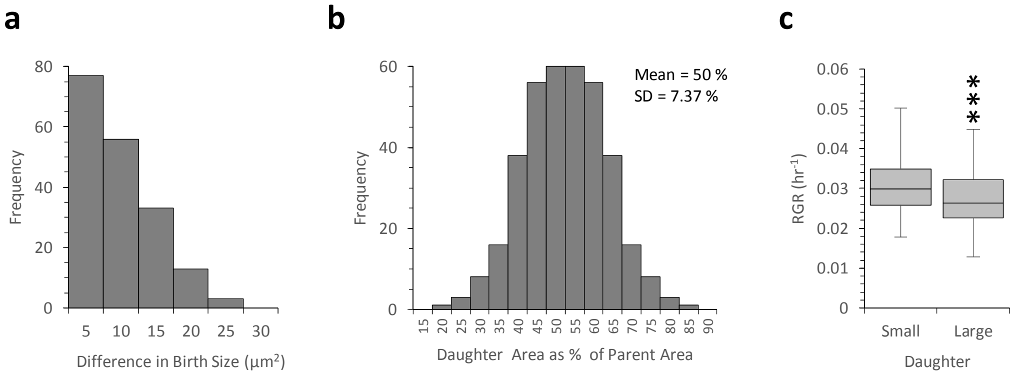

Supplementary Figure 2: Analysis of unequal divisions

a) Frequency of differences in cell area at birth. Difference in birth size is calculated as the area of the larger daughter at birth minus the area if the smaller of daughter at birth. n = 182. Cells are taken from the central zone.

b) Frequency distribution of division ratios for 364 divisions observed in the central zone. Division ratio calculated as birth size of daughter as a percentage of the division size of the parent.

c) Comparison of the mean RGR of the large and small daughters from 182 divisions in the central zone. RGR is calculated across the length of the cell cycle. (t-test, $t = -4.9203$, $df = 359.362$, $p < 0.001$). Note that the difference in mean RGR (<0.01 hr^{-1}), is smaller in magnitude than the difference in RGR in between young and old primordia (~ 0.05 hr^{-1}). Boxes represent the interquartile range, whiskers represent total range. *, **, *** indicate significance at the 0.05, 0.01 and 0.001 levels respectively.

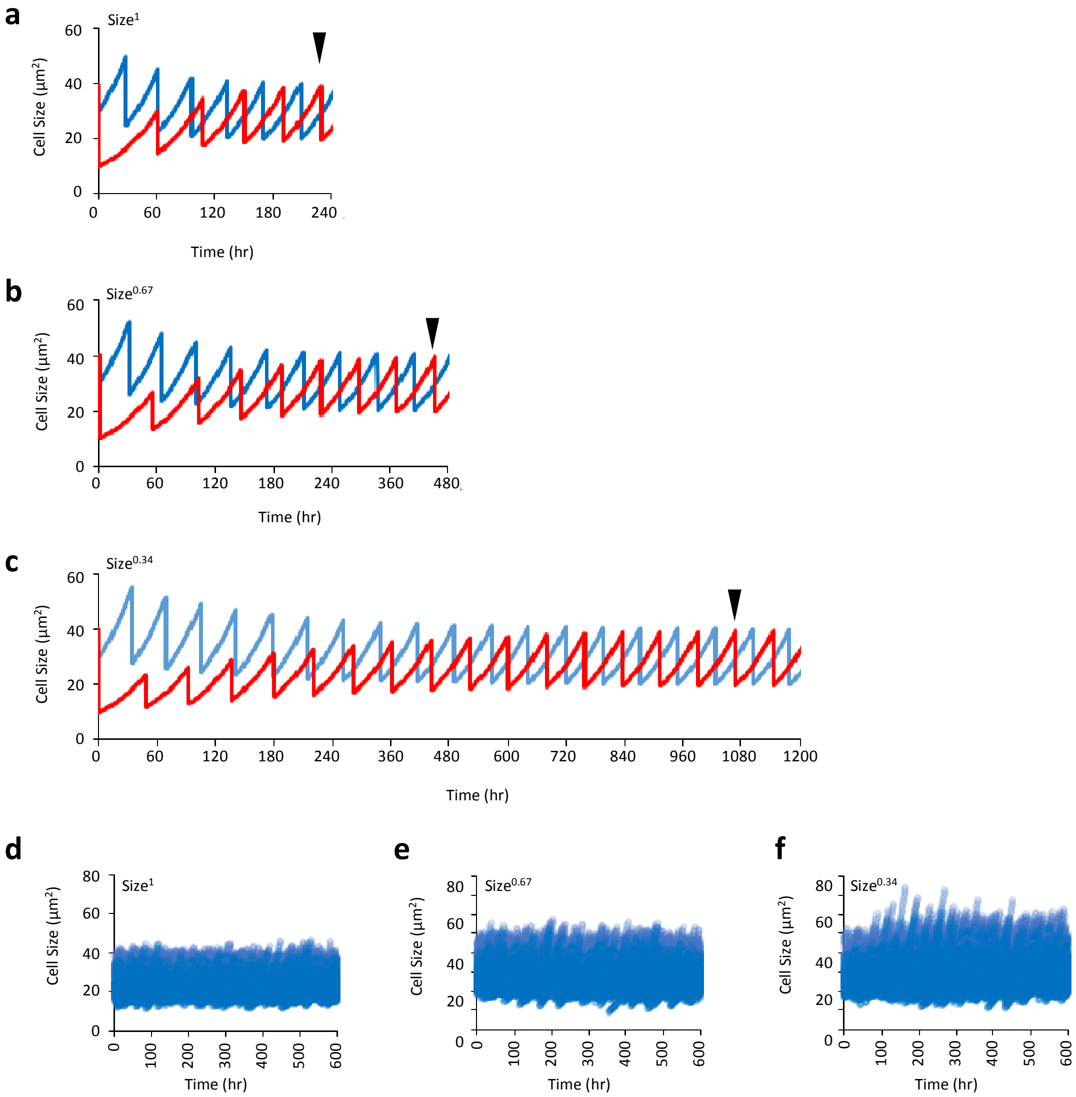

Supplementary Figure 3: Effect of altering the scaling between *pCDK* and cell size

a-c) To investigate whether the manner in which *pCDK* scales with size affects cell size control the model was run with *pCDK* is proportional to (a) $Size^{1}$ (equivalent to pCDK scaling with the same dimensions), (b) $Size^{0.67}$ (equivalent to a 2D parameter scaling with a 3D parameter or 1D parameter scaling with a 2D parameter) and (c) $Size^{0.34}$ (equivalent to a 1D parameter scaling with a 3D parameter). Simulations were run without variation in *g* and *d* and were designed to test the effect of an uneven division. The simulation was initiated with a large cell (blue) and a small cell (red). Graphs show cell size over time, representing a period of at least six division cycles. Peaks correspond to cell size at division. Black arrow heads indicate the time after which both lineages show the same size at division. All simulations show cell size control, but $Size^{0.67}$ and $Size^{0.34}$ simulations require a greater number of cycles to return to a steady state following an uneven division.

d-f) Results of model simulations of populations of cell where *pCDK* is proportional to $Size^{1}$, $Size^{0.67}$ and $Size^{0.34}$ respectively. Simulations were run with variation in *g* and *d* according to observed values. The population was initiated with 100 asynchronously cycling cells. All populations show cell size control, but the distribution of cell sizes within the populations are larger when $Size^{0.67}$ and $Size^{0.34}$ are used.

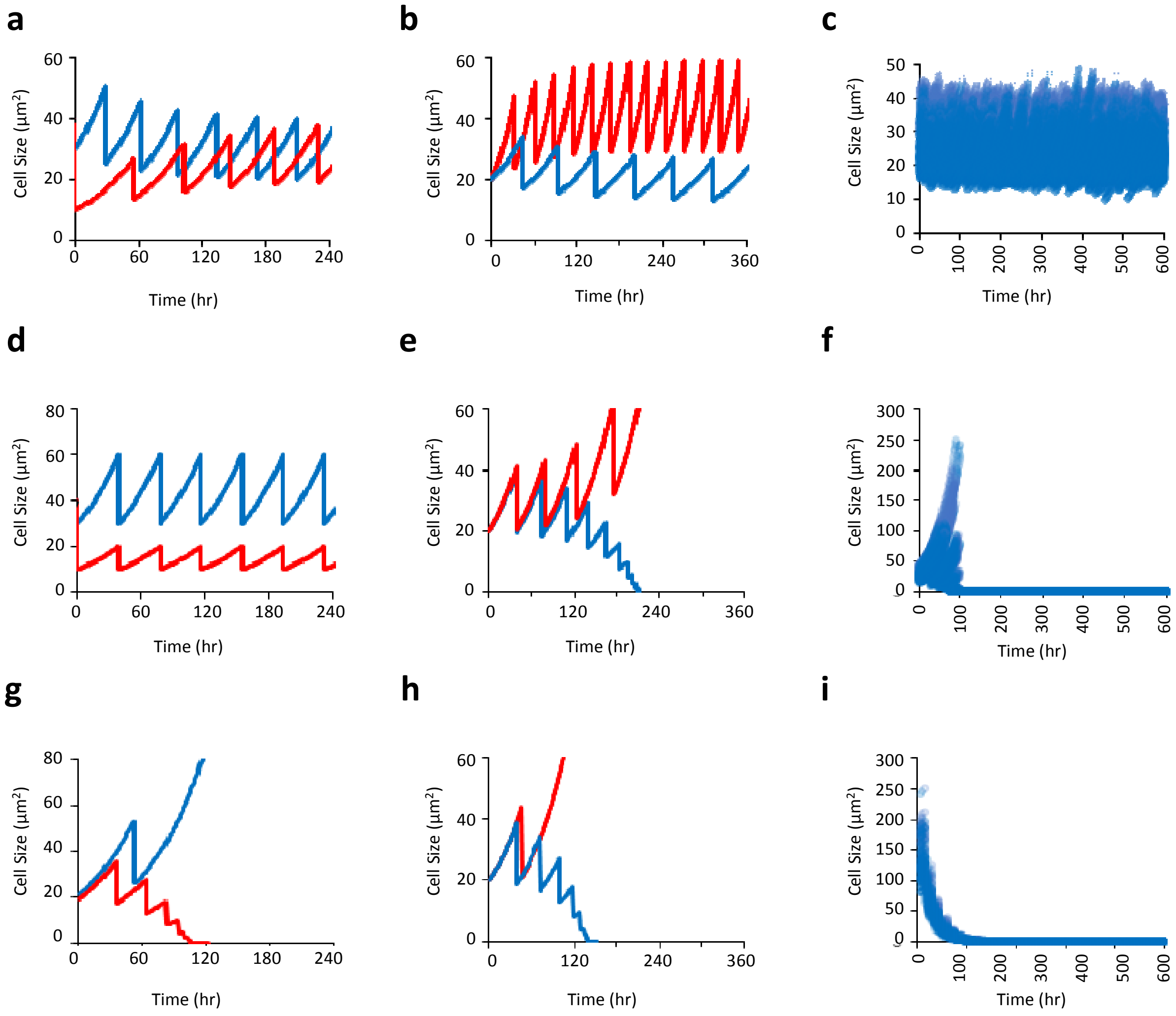

Supplementary Figure 4: Exploration of links between cell size and cell cycle progression.

a,b) Results of model simulation with T_{Division} inversely proportional to cell size. Simulations were run without variation in *g* and *d* and were designed to test the effect of an uneven division (a) or a change in RGR (b). In the uneven division test the simulation was initiated with a large cell (blue) and a small cell (red). In the RGR test, the simulation was run with increased (red) or decreased (blue) relative growth rate *g*. Graphs show cell size over time, representing a period of at least six division cycles. Peaks correspond to cell size at division.

c) Results of model simulation with T_{Division} inversely proportional to cell size, including variation in *g* and *d* according to observed values. The population is initiated with a population of 100 asynchronous cells.

d,**e**) Results of model simulation with $pCDK$ inversely proportional to cell size. Simulations were run without variation in g and d and were designed to test the effect of an uneven division (d) or a change RGR (e). In the RGR test, the simulation was run with increased (red) or decreased (blue) relative growth rate g. Graphs show cell size over time, representing a period of at least six division cycles. Peaks correspond to cell size at division.

f) Results of model simulation with $pCDK$ inversely proportional to cell size, including variation in g and d according to observed values. The population is initiated with a population of 100 asynchronous cells. In this model large cells have a longer cell cycle than small cycles. This results in lineages of large cells that continue increasing in size with successive generations and lineages of small cells that continue getting smaller (0-100 time iterations), however, overtime, the increasingly short cycles of small cells mean that the small lineages outcompete the large lineages (100 iterations onwards).

g,**h**) Results of model simulation with T_{Division} proportional to cell size. Simulations were run without variation in g and d and were designed to test the effect of an uneven division (g) or a change RGR (h). In the RGR test, the simulation was run with increased (red) or decreased (blue) relative growth rate g. Graphs show cell size over time, representing a period of at least six division cycles. Peaks correspond to cell size at division. Note that even a very small asymmetry in division (2 μm^2 difference in birth size) is sufficient to result in unstable conditions.

i) Results of model simulation with T_{Division} proportional to cell size, including variation in g and d according to observed values. The population is initiated with a population of 100 asynchronous cells. In this model large cells have a longer cell cycle than small cycles and small lineages very rapidly outcompete large cell lineages.

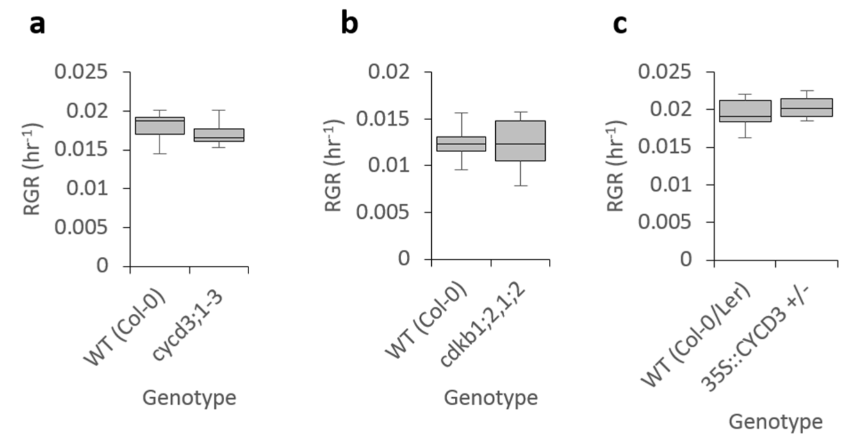

Supplementary Figure 5: Mean RGR of cells in stems with altered production of CDK activity

a) Comparison of mean RGR of central zone cells in WT (Col0) and *cycd3;1-3* triple mutant stems. Data represent means from 9 and 7 plants respectively. (t-test, t = -0.7763, df = 13.196, p = 0.4513).

b) Comparison of mean RGR of central zone cells in WT (Col0) and *cdkb1;1/1;2* double mutant stems. Data represent means from 13 and 11 plants respectively. (t-test, t = -0.0226, df = 12.002, p = 0.9824).

c) Comparison of mean RGR of central zone cells in WT (Col0/Ler) and *35s::CYCD3;1 +/-* stems. Data represent means from 5 and 7 plants respectively. (t-test, t = 0.7818, df = 6.742, p = 0.4609).

Boxes represent the interquartile range, whiskers represent total range.

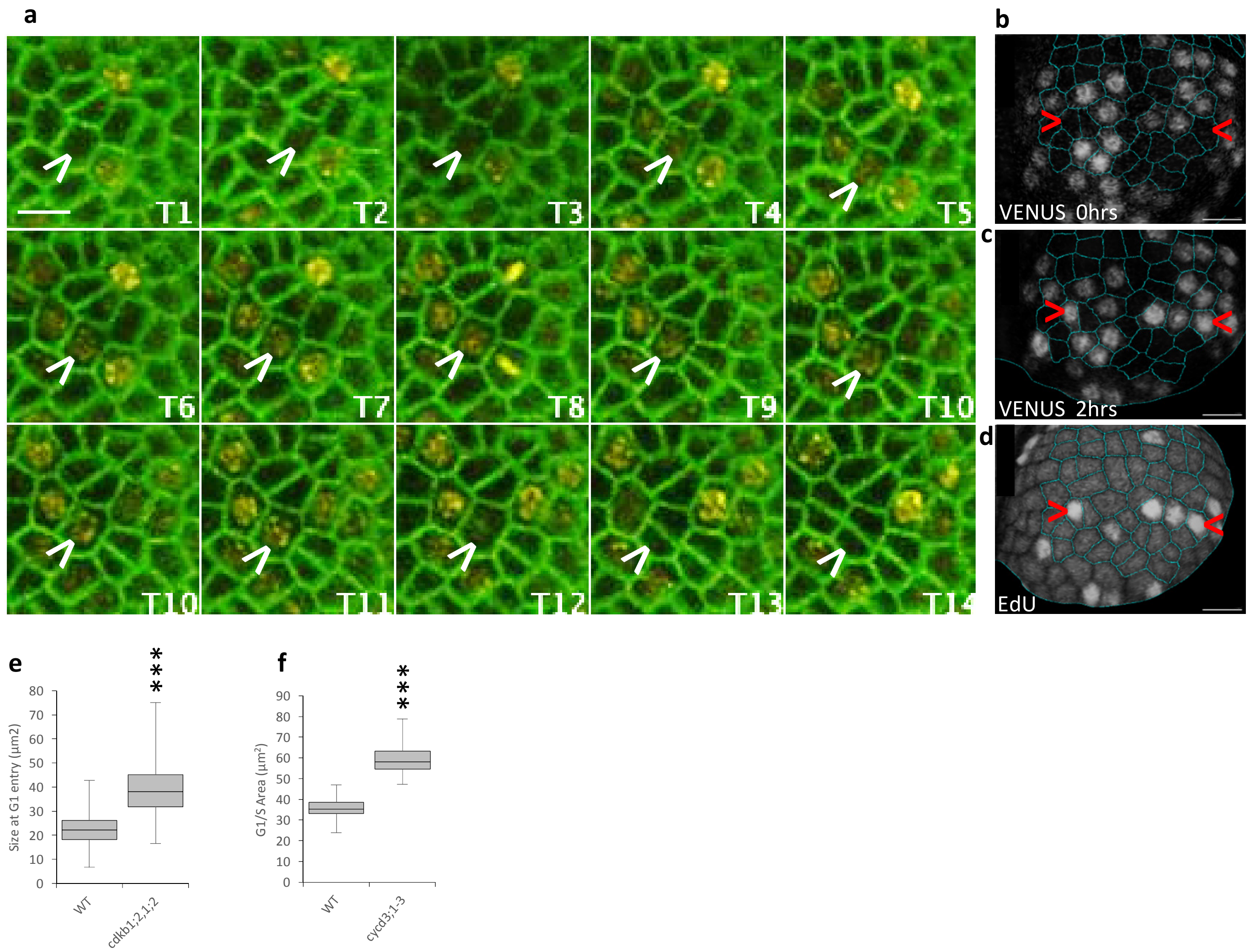

Supplementary Figure 6: Characterisation of timing of *H4::DB-VENUS* expression.

a) Time course images showing dividing cells in a young organ primordia of plants carrying the *H4::DB-VENUS* construct over seven hour period. VENUS signal is shown in yellow. Cell membranes are shown in green. White arrows indicate the same dividing cell throughout the time course. Images are taken at 30 minute intervals. Note that *H4::DB-VENUS* is detected before division, but disappears after the formation of a new wall. Scale bar represents 10 µm.

b-d) Expression of *H4::DB-VENUS* at the beginning (**b**) and end (**c**) of the EdU feeding period and EdU detection in the same cells (**d**). Cell boundaries from segmentations based on Fm4-64 and PI staining respectively are shown in blue. Red arrows indicate cells where *H4::DB-VENUS* is switched on during the EdU feeding period. Note that incorporation of EdU is also detected in these cells, indicating that there is no significant lag between *H4::DB-VENUS* expression and the initiation of DNA synthesis. Scale bars represent 10 µm.

e) Distribution of cell sizes at G1 entry in WT (Col-0) and *cdkb1;1/1;2*. Data represent cells from 13 and 11 plants respectively. The larger size of *cdkb1;1/1;2* cells at birth means cells are larger on entering G1 than WT cells. (Effect Size = 16.40 µm2 ± 0.52, $p < 0.001$).

f) Distribution of cell sizes at the G1/S transition in WT (Col-0) and *cycd3;1-3*. The larger size of cells at the G1/S transition means *cycd3;1-3* cells are larger than wild type on entering S-G2-M. Data represent cells from 9 and 7 plants respectively. (Effect Size = 23.27 $\mu m^2 \pm 1.31$, $p < 0.001$).

Boxes represent interquartile range, whiskers represent total range. *, **, *** indicate a significance at the 0.05, 0.01 and 0.001 levels respectively.

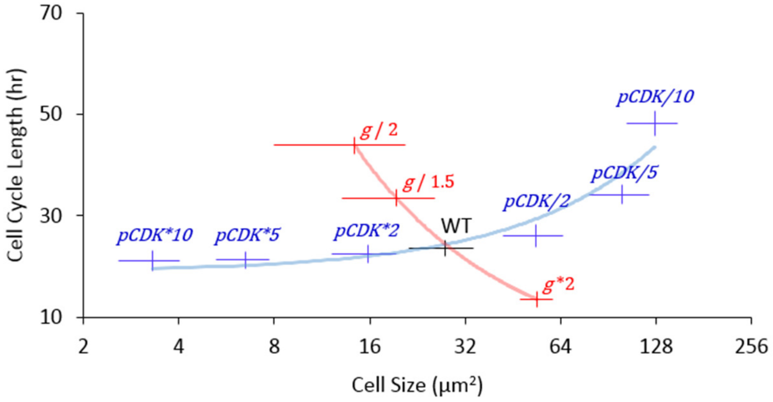

Supplementary Figure 7: Model predictions with RGR dependent on cell size at birth.

Model simulations were initiated with a population of 100 asynchronous cells. The effect of altering RGR (red) and *pCDK* (blue) on the distribution of cell sizes and cell cycle is shown. T_{Division} is unaltered. Relative fold changes in parameter values relative to WT values are indicated on the graphs. In comparison to Figure 4j in which cell size but not cell cycle length are altered in response to *pCDK*, both cell size and cell cycle length are affected in this version of the model. Note also that when *pCDK* is changed in this model, there is a positive relationship between cell size and cell cycle length. Data points represent means, error bars represent standard deviation.

Supplementary Table 1: Values and units of variables used in cell cycle model simulations

Parameter	Value	Units	Standard Deviation	Reference
g	0.018	hr^{-1}	0.0018	
d	50	%	7.37	
T_{Division}	2200	Arbitrary Units	-	[1]
$T_{\mathrm{DivisionS}}$	Variable	Arbitrary Units per μm^2		
$T_{\mathrm{G1/S}}$	1600	Arbitrary Units	-	[1]
$T_{\mathrm{G2/M}}$	2200	Arbitrary Units	-	[1]
$pCDK$	See Supplementary Table 2	Arbitrary Units per hr		
$pCDK_{\mathrm{S}}$	See Supplementary Table 2	Arbitrary Units per hr		
$pCDK_{\mathrm{M}}$	See Supplementary Table 2	Arbitrary Units per hr		

Supplementary Table 2: *pCDK* values used in one and two transition models of the cell cycle

Model	Parameter	Value (Arbitrary Units.hr^{-1})
One Transition Model:		
No Size Dependency	*pCDK*	57.14
pCDK Size Dependent	*pCDK*	2
T_{Division} Size Dependent	*pCDK*	1.46
Two Transition Model:		
$pCDK_{\text{S}}$ Size Dependent	$pCDK_{\text{S}}$	2.9
	$pCDK_{\text{M}}$	140
$pCDK_{\text{M}}$ Size Dependent	$pCDK_{\text{S}}$	70
	$pCDK_{\text{M}}$	4
$pCDK_{\text{S}}$ & $pCDK_{\text{M}}$ Size Dependent	$pCDK_{\text{S}}$	2.8
	$pCDK_{\text{M}}$	4
